# The Manifestation of the Dual ROS-Processing and Redox Signaling Roles of Glutathione Peroxidase-like Enzymes in Development of *Arabidopsis* Seedlings

**DOI:** 10.3390/antiox14050518

**Published:** 2025-04-25

**Authors:** Krisztina Bela, Bernát Tompa, Riyazuddin Riyazuddin, Edit Horváth, Krisztián Jász, Ádám Hajnal, Sajid Ali Khan Bangash, Ágnes Gallé, Jolán Csiszár

**Affiliations:** 1 Department of Plant Biology, Faculty of Science and Informatics, University of Szeged, H-6726 Szeged, Hungary; belakriszti88@gmail.com (K.B.); tompa_bernat@yahoo.com (B.T.); riyazkhan24992@gmail.com (R.R.); horvath.edit.03@szte.hu (E.H.); gyorsabb6@gmail.com (K.J.); hajnal.adam.barnabas@szte.hu (Á.H.);; 2 Doctoral School of Biology, Faculty of Science and Informatics, University of Szeged, H-6726 Szeged, Hungary; 3 Bioengineering Institute, Miguel Hernández University, 03202 Elche, Spain; 4 Institute of Biotechnology and Genetic Engineering, The University of Agriculture, Peshawar 25130, Pakistan; sajidbangash@aup.edu.pk

**Keywords:** abiotic stress, *Arabidopsis thaliana*, glutathione peroxidase-like enzymes, reactive oxygen species, redox potential, roGFP2, root growth

## Abstract

Plant glutathione peroxidase-like (GPXL) enzymes are thiol-based peroxidases that reduce H_2_O_2_ or hydroperoxides to water or alcohols using electrons principally from thioredoxin. *Arabidopsis thaliana* possesses eight isoenzymes (AtGPXL1−8) located in different plant organelles and have various roles in redox-dependent processes. The determination of the redox potential of 6-day-old T-DNA insertional mutants (*Atgpxl1*–*Atgpxl8*) using a cytosolic redox-sensitive fluorescent probe (roGFP2) uncovered more oxidized redox status in the shoot and/or root of the untreated mutants, except for *Atgpxl5*. To investigate the involvement of AtGPXLs in the growth and abiotic stress responses of seedlings, the 4-day-old *Atgpxls* were exposed to salt and osmotic stresses for two weeks. The evaluation of the reactive oxygen species (ROS) levels of untreated 18-day-old plants using fluorescent microscopy revealed the elevated accumulation of total ROS in the shoots and, in some cases, the roots of the mutants. Regarding the growth of roots, both the length of primary roots and/or the number of lateral roots were affected by the mutation of *AtGPXLs*. A strong negative correlation was observed between the ROS level of wild type shoots and the development of lateral roots, but it was altered in mutants, while in the case of *Atgpxl1*, *Atgpxl5*, and *Atgpxl7* seedlings, it disappeared; in other mutants (*Atgpxl4*, *Atgpxl6*, and *Atgpxl8*), the correlation became stronger. Our analysis underpins the discrete role of AtGPXL enzymes in controlling the growth and development of plants by fine tuning the ROS contents and redox status in an organ-specific way. Differences in root phenotype and metabolic activity between *Atgpxl* mutants and wild type plants highlight the essential role of AtGPXLs in ROS processing to support growth, which is particularly evident when one GPXL isoenzyme is absent or its activity is reduced, both under normal and abiotic stress conditions.

## 1. Introduction

Adverse environmental conditions may increase the production of reactive oxygen species (ROS), such as superoxide radicals (O_2_^•−^), hydrogen peroxide (H_2_O_2_), hydroxyl radicals (OH^•^), and singlet oxygen (^1^O_2_). Although the elevated level of ROS may induce detrimental oxidation of macromolecules, a lot of evidence supports that a change in endogenous oxidant levels can fulfill signaling functions regulating normal plant growth and responses to stresses. ROS play a central role in integrating external and intracellular signals and, in addition to stress responses, are important regulators of plant growth and development [1,2,3,4]. Although the role of ROS in signal transduction is widely accepted, the precise signaling mechanisms leading to specific responses remain poorly understood.

To keep ROS levels tightly controlled and minimize ROS-derived impairments, different non-enzymatic antioxidants (such as ascorbate, glutathione, carotenoids, and tocopherols) and ROS-processing enzymes, including glutathione peroxidases, have evolved in aerobic organisms [4,5]. The glutathione peroxidase (GPX or GPx) enzyme family is found in most eukaryotic organisms. However, they have diverged early in evolution and diversified independently in terms of structure, catalytic mechanism, and substrate usage across kingdoms [6]. Robust phylogenetic and sequence analyses suggested that all GPX-encoding genes likely evolved from a monomeric common ancestor. This ancestral enzyme was thought to be promiscuous with respect to reducing agents and substrates, resembling bacterial GPXs [6]. They might possess peroxidatic cysteins in their active site, and their activity is considered to be the reduction in lipid peroxides, as they can be observed in the fungal, plant, and non-seleno animal proteins [6]. In the most studied mammals, the GPXs are pivotal ROS-eliminating antioxidant enzymes that can reduce H_2_O_2_ and organic peroxide substrates to water or corresponding alcohols using reduced glutathione (GSH) as electron donors [5,7]. In addition to participating in the maintenance of membrane integrity, they can oxidase cysteine-containing proteins involved in signaling, such as phosphatases, kinases, and transcription factors, and thus induce or regulate different pathways. GPXs are key players in many biological processes, such as fertility, anti-inflammatory-, and anti-carcinogenesis-associated routes [5,7].

Among plant glutathione peroxidases, the most well studied are those of *Arabidopsis thaliana* [8]. *A. thaliana* is a widely used model organism in plant physiology and genetics. It has a relatively small genome, a short lifecycle, and produces numerous self-progenies. Due to the ease of efficient genetic transformation by *Agrobacterium tumefaciens*, a large number of T-DNA insertion mutants are available from stock centers that can be used for the identification of mutations in particular genes, thus serving as excellent reverse genetic resources. Gene functional information from *Arabidopsis* can be applied to other plant species [9].

Phylogenetic analysis of a total of 169 fully sequenced GPX protein sequences from various species across different kingdoms and phyla were analyzed to determine their phylogenetic relationships [10]. Plant GPXs were categorized into five groups based on their clustering in the phylogenetic tree. Meanwhile, the animal GPXs clustered into a sixth group, exhibiting some evolutionary connection to plant GPXs. Comparative analysis revealed highly conserved motifs and exon–intron structures within each group [10,11], suggesting structural and functional preservation through evolution. The *Arabidopsis* GPXs were distributed across all five plant-specific clusters alongside other plant GPXs [10], supporting the notion that these enzymes share key characteristics with each other but differ significantly from well-known mammalian GPXs.

In contrast to the animal GPXs, plant isoenzymes are selenium-independent monomeric proteins containing cysteine in their catalytic site; therefore, they show lower peroxidase activity than the animal enzymes, and most of them prefer thioredoxin (TRX) as an electron donor rather than GSH [10,11]. Since plant glutathione peroxidases are more similar to mammalian GPX4, which is a membrane-associated phospholipid hydroperoxidase [5], the proposed main function of plant enzymes has been the reduction in lipid peroxides and the maintenance of membrane integrity [11]. Although several studies have shown that they participate in the conversion of lipid hydroperoxides to less toxic molecules, their specific features also suggest involvement in other key processes, such as signaling and oxidative protein folding [11,12]. Due to the structural similarity of plant GPXs to animal GPXs, yet differing enzymatic activities and substrate specificities, Meyer and his colleagues adopted the GPX-like (GPXL) nomenclature for the *Arabidopsis thaliana* isoforms (AtGPXLs) to prevent misinterpretation regarding their function [13]. We now apply the GPXL abbreviation in all references to previously published information about plant glutathione peroxidases. Regarding the glutathione peroxidases, *Arabidopsis* possesses eight isoenzymes (AtGPXL1–AtGPXL8) located in different plant organelles and having various roles in redox-dependent processes [8,13].

The transcription levels of plant *GPXLs* were increased by various environmental stress conditions, and numerous scientific reports provided evidence for their importance in different stress responses [8,10,11,14,15]. The overexpression of *GPXL* genes led to higher tolerance against abiotic stress factors in tomato [16,17], rice [18], tobacco [19], *A. thaliana* [20,21,22], and *Salvia miltiorrhiza* [23]. Moreover, GPXLs are involved in the development and growth of plants. Passaia et al. [24] compared phenotypes of 4-week-old *Arabidopsis Atgpxl* mutants and observed that although their shoot was largely similar to wild type plants, minor differences were found in the number of rosette leaves and lateral roots in the *Atgpxl2*, *Atgpxl3*, *Atgpxl7*, and *Atgpxl8* mutants. Connections among the AtGPXLs and phytohormones such as auxin, abscisic acid, and strigolactone hormones were suggested, thereby demonstrating the importance of AtGPXLs in hormone-mediated regulation, especially of lateral root development. These authors also proposed that GPXLs may be required to mediate glutathione and reduced thioredoxin functions in roots that impact lateral root production or growth [24]. GSH and TRX are the main components of cellular redox homeostasis and are important for the processes that determine the root architecture [25]. In the case of an acute shortage of glutathione, more TRX is used as an electron donor compared to GSH, suggesting a possible connection between the GSH and TRX pools [26].

The involvement of glutathione (γ-Glu-Cys-Gly) and GSH-related antioxidant enzymes in redox homeostasis and signaling came to the forefront in the last decades [4,11,27,28]. Glutathione is classically considered one of the main antioxidants with low molecular weight, playing a crucial role in ROS detoxification in cooperation with ascorbate in the Foyer–Halliwell–Asada pathway and serving as an electron donor to diverse antioxidant enzymes [4]. Reactive oxygen and nitrogen species, ROS-processing enzymes, and other molecules with antioxidant activity, along with their redox states, all contribute to the overall redox homeostasis of the cell [29]. Now, it is widely accepted that the ratio of reduced and oxidized glutathione (GSH/GSSG) and the glutathione half-cell reduction potential (*E*_GSSG/2GSH_), which depends on the absolute glutathione concentration and the [GSH]:[GSSG] ratio, are effective markers of redox homeostasis [30,31]. Enhanced ROS production temporarily shifts the redox potential to more oxidized values [12], which was proven in vivo by genetically encoded redox probes [32,33].

Genetically encoded biosensors are fluorescent proteins that change their properties specifically in response to a physiological parameter [34]. Among them, roGFP2 (reduction–oxidation sensitive green fluorescent protein 2) is applied the most frequently for examining the redox state in plants (e.g., [13,26,34,35,36,37]). In this redox sensor, specific amino acids of wild type fluorescent protein that influence chromophore structure were replaced, and two Cys residues were introduced. The redox status of the Cys pair is dependent on the GSH/GSSG redox state, and the rapid equilibration between them is facilitated by a linked glutaredoxin (GRX) molecule [32,38,39]. The oxidation of roGFP induces a change in the protonated state of the chromophore: it increases fluorescence intensity at the excitation band associated with the protonated form (400 nm) and decreases fluorescence intensity at the excitation band corresponding to the anionic form (490 nm), enabling ratiometric fluorescence readout. From the fluorescence intensities detected at the two excitation wavelengths, we can calculate the glutathione-dependent redox potential [32,39].

Being thiol peroxidases that harbor redox-active Cys in their active site, GPXL proteins are very sensitive to oxidation. Using GSH and/or TRX as a reductant, the GPXLs may control the redox status of these main redox compounds; also, there may be an important connection between them. They can modify the thiol/disulfide balance and activity of other proteins and transcription factors [40,41]. They not only were considered to function as redox sensors but also as a link between ROS and functional redox signaling [12,24,40,41,42]. As efficient ROS-processing antioxidant enzymes, GPXLs are involved in controlling ROS levels and shaping ROS gradients. They play a role in maintaining the stem cell niche, triggering differentiation in the shoot and root apical meristems (SAM and RAM, respectively), and ensuring proper zygote/embryo development [43].

In the present study, we have introduced the roGFP2 redox probe into the *Atgpxl1*–*8* mutants to detect the redox status of seedlings. Measuring the glutathione redox potential was conducted on 6-day-old seedlings. Our aim was to analyze the involvement of AtGPXLs in the growth and development of roots of seedlings and their role in the maintenance of ROS levels in shoots and roots. In addition to the evaluation of O_2_^•−^ and the overall level of intracellular ROS content (total ROS) by fluorescent dyes, the vitality (metabolic activity) of in vitro grown roots of *Atgpxl1*–*8* mutants were investigated under control conditions and after 14-day-long treatment with mild salt (50 and 100 mM NaCl) and osmotic stresses (100 and 200 mM mannitol). It was concluded that AtGPXLs have a fine-tuning role in processing ROS in seedlings both under control and abiotic stress conditions. Although some other antioxidant mechanisms can be activated, especially in case of stronger stresses, these are not capable of suppressing the ROS levels, and thus a mutation in one of the *AtGPXL* genes may cause a change in overall redox status.

## 2. Materials and Methods

### 2.1. Plant Material and Growth Conditions

*Arabidopsis thaliana* (L.) ecotype Columbia (Col-0) as a wild type and T-DNA insertional mutants of the eight glutathione peroxidase-like genes (*AtGPXL1*–*8*) were used. The T-DNA insertional mutant lines were obtained from the Nottingham Arabidopsis Stock Centre (NASC) (*Atgpxl1* [AT2G25080]: SALK_128885C; *Atgpxl2* [AT2G31570]: SALK_082445C; *Atgpxl3* [AT2G43350]: SALK_071176C; *Atgpxl4* [AT2G48150]: SAIL_623_F09; *Atgpxl5* [AT3G63080]: SALK_076628C; *Atgpxl6* [AT4G11600]: WiscDsLox321H10; *Atgpxl7* [AT4G31870]: SALK_072007C; *Atgpxl8* [AT1G63460]: SALK_127691C), and the seeds of homozygous genotypes were used. According to our previous results, *Atgpxl1*, -*2*, -*5*, and -*6* are knockdown mutants, while *Atgpxl3*, -*4*, -*7*, and -*8* are knockout mutants [44].

Seeds were sown on ½ MS media [45] and were pre-grown in a growth chamber (Fitoclima S 600 PLH, Aralab, Rio de Mouro, Portugal) at 21 °C under 100 μmol m^−2^ s^−1^ photon flux density with a 10 h day and 14 h night photoperiod. The redox potential was measured in 6-day-old seedlings. To estimate the growth parameters and ROS levels under different abiotic stresses, the 4-day-old seedlings were transferred to ½ MS media supplemented with 50 mM and 100 mM NaCl or 100 mM and 200 mM mannitol in square Petri plates and placed vertically in the same chamber for 2 weeks to check differences in long-term responses.

### 2.2. Analysis of the Redox Potential by Ratiometric Measurements of the roGFP2 Fluorescent Probe

To determine the redox potential of seedlings, a fusion protein of human glutaredoxin1 (GRX1) and roGFP2 protein with cytosolic localization (c-roGFP2) was used [31,46]. This vector, kindly provided by Prof. Dr. A. Meyer, had been introduced into wild type (Col-0) *Arabidopsis* earlier, as published in [35]. The redox sensor was introduced into *Atgpxl1*–*8* mutants by crossing, and homozygous mutants were established. Fluorescence measurements were performed with a confocal laser scanning microscope (Olympus Fluoview FV1000, Olympus Life Science Europe GmbH, Hamburg, Germany) in the cotyledon and the apical meristematic region (proximal meristem) of 6-day-old seedlings. Excitation wavelengths were 405 nm and 488 nm, and the fluorescence was detected between the 505–530 nm emission wavelength [35].

The glutathione redox potential (*E*_GSH_) was calculated using the following formula [31]:

$$E_{GSH}=E_{roGFP2}^{0}-\left(\frac{2303\times R\times T}{z\times F}\right)\times {log}_{10}\frac{1-{OxD}_{roGFP2}}{{OxD}_{roGFP2}}$$

where $E_{roGFP2}^{0}$, the midpoint potential of roGFP2, is −272 mV at 30 °C pH 7; *R* is the gas constant (8.315 J K^−1^ mol^−1^); *T* is the absolute temperature (298.15 K); *z* is the number of transferred electrons (2); and *F* is the Faraday constant (96,485 C mol^−1^). The degree of oxidation (OxD) of roGFP2 was as follows:

$${OxD}_{roGFP2}=\frac{R-R_{red}}{\left(\frac{I_{488red}}{I_{488ox}}\right)\times \left(R_{ox}-R\right)+\left(R-R_{red}\right)}$$

where *R* is the ratio of excitation at 405/488 nm; *R_red_* is the ratio of the fully reduced form using 10 mM dithiothreitol; *R_ox_* is the ratio of the fully oxidized form using 20 mM H_2_O_2_; *I*_488*ox*_ is the intensity at 488 nm for the fully oxidized form; and *I*_488*red*_ is the intensity at 488 nm for the fully reduced form.

### 2.3. Measurements of Root Parameters

Root length and the number of lateral roots of the Col-0 and *Atgpxl1*–*8* mutants were analyzed using Fiji (ImageJ2 version 2.1.0/1.53c, 2020 and release) software [47] after scanning the square plates, as described earlier in [22]. Lateral roots were counted, and lateral root density (LRD) was calculated by dividing the number of visible lateral roots by the primary root length [24].

### 2.4. Detection of the Vitality, Superoxide Radical, and Total ROS Levels in Roots and Leaves

*A. thaliana* seedlings were incubated for 15 min in 3 mL 10 μM fluorescein diacetate (FDA) staining solution (prepared in 10/50 mM MES/KCl buffer, pH 6.15) to determine cell vitality [35] and were then washed four times with MES/KCl before detection.

For a visualization of the total ROS level, 2′,7′-dichlorodihydrofluorescein diacetate (H_2_DCFDA) fluorescent dye was used. Seedlings were incubated in 3 mL 50 μM H_2_DCFDA staining solution (prepared in 50 mM sodium phosphate buffer, pH 7.5) for 30 min and washed twice with the buffer before detection [48].

Dihydroethidium (DHE) in Tris–HCl (10 mM, pH 7.4) buffer was used to detect superoxide (O_2_^•−^) radical levels in roots (with the available filter, the detection in the shoots was not possible). Seedlings were incubated in darkness for 30 min with 3 mL 10 μM DHE, and then the samples were washed twice with buffer before detection [35].

A Zeiss Axiowert 200 M microscope (Carl Zeiss, Jena, Germany) equipped with a high-resolution digital camera (Axiocam HR, HQ CCD, Carl Zeiss, Jena, Germany), filter set 10 (excitation 535–585 nm, emission 600–655 nm) for FDA and H_2_DCFDA, and filter set 9 (exc.: 450–490 nm, em.: 515–∞ nm) for DHE was used to detect fluorescence in plants.

The intensities of fluorescein-2′,7′-dichlorofluorescein and oxyethidium fluorescence were quantified on digital images using Axiovision Rel. 4.8 software in the proximal meristem of the roots in a circle with a 50 μm radius [35] or in the middle of the leaves in a circle with a 150 μm radius. The measurements were performed in three independent experiments (n ≥ 15) with the same microscopic settings.

### 2.5. Statistical Analysis

Experiments were conducted at least three times, except for redox potential measurements, where in some cases, only two biological replicates were performed due to the limited number of homozygous roGFP2-expressing lines. Other values are presented as the mean ± standard error (SE), with n ≥ 45. For redox potential measurements, data are shown as the mean ± standard deviation (SD), with n ≥ 3.

Statistical analysis was performed using SigmaPlot 12.0 (SigmaPlot, Milan, Italy). After analysis of variance (ANOVA), Duncan’s multiple comparisons test was conducted. Means were considered significantly different at *p* ≤ 0.05.

The correlation analysis was performed on data including measured growth parameters, ROS levels, and metabolic activity of 18-day-old seedlings under control conditions and two weeks after treatment with 50/100 mM NaCl or 100/200 mM mannitol in *A. thaliana* wild type (Col-0) and *Atgpxl1*–*8* mutant seedlings. (*E*_GSH_ determination was conducted on 6-day-old seedlings, as the lower number of cell layers allows for more precise detection of ratiometric roGFP2 fluorescence.)

The correlation was estimated based on total ROS levels and metabolic activities in shoots and roots and separately with superoxide radical anion levels in roots, total root length, the number of lateral roots, and lateral root density. To assess the relationships between these variables, we calculated Pearson’s correlation coefficients using the R program (version 3.6.2, R Core Team, 2019) [44].

The correlation coefficient values ranged from +1 to −1, where values close to +1 or −1 indicated a strong positive or negative relationship between the variables, respectively. As the coefficient approaches 0, the relationship between the two variables weakens.

## 3. Results

### 3.1. The Redox Status of 6-Day-Old Atgpxl Mutant Seedlings Are More Oxidized Compared to the Wild Type

**Figure 1:**
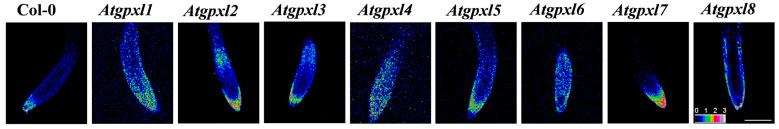
Representative images of the ratiometric analysis of redox status (ratio of fluorescence intensity at 405 and 488 nm based on the given color scale) of 6-day-old *Arabidopsis* Col-0 (wild type) and *Atgpxl1*–*8* mutant roots expressing a cytosolic roGFP2 fluorescent probe using an Olympus Fluoview FV1000 confocal microscope. The white scalebar is equal to 100 µm.

The determination of the redox potential, using the redox-sensitive fluorescent probe (roGFP2) in vivo, uncovered a more oxidized redox state in most of the untreated mutants (Figure 1, Table 1). In leaves, the highest oxidized redox status was found in *gpxl1* and -*7* seedlings, and a ca. 25 mV difference in the redox status was detected between these mutants and the wild type plants. In roots, more than a 20 mV increase was detected in the case of *gpxl1* and -*4* mutants. Interestingly, in the cotyledons, the redox potential of *gpxl4* and -*5* mutants was comparable to the wild type plants; moreover, in the roots, the *E*_GSH_ of *gpxl3*, -*5*, and -*7* did not differ significantly from Col-0 (Table 1). This may indicate that other mechanisms might complement the default of some AtGPXL isoenzymes in mutants or that the coding enzymes might have no relevant function in the maintenance of redox homeostasis and ROS processing in seedlings.

### 3.2. Several Atgpxl Mutants Had Longer Primary Roots than the Wild Type Controls, and 50 mM NaCl Promoted the Growth of All Genotypes Compared to the Untreated Col-0 Primary Roots

**Figure 2:**
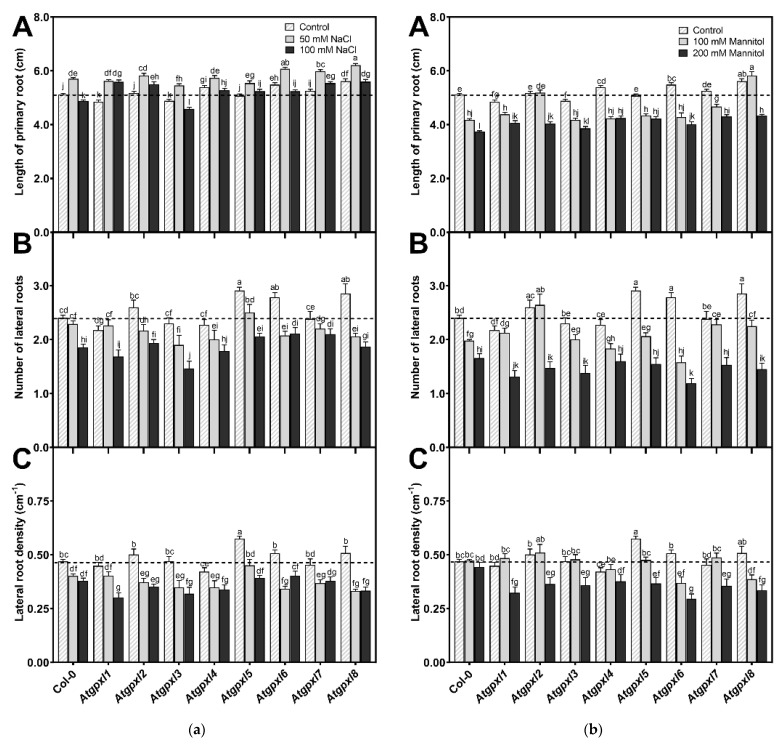
Root growth parameters of the 18-day-old *Arabidopsis* Col-0 and *Atgpxl1*–*8* mutant seedlings. (**a**) Length of primary root (A), number of lateral roots (B), and lateral root density (C) under control conditions and after two weeks of 50 mM and 100 mM NaCl treatments. (**b**) Length of primary root (A), number of lateral roots (B), and lateral root density (C) after two weeks of 100 mM and 200 mM mannitol treatments. Dashed lines show the values of the untreated wild type to highlight the differences compared to those data. Data are the mean ± SE, n ≥ 45, and were analyzed using one-way ANOVA followed by Duncan’s multiple range test. Different letters represent data considered statistically significant at *p* ≤ 0.05.

The involvement of AtGPXLs in plant growth under control and abiotic stress responses was investigated after exposing the 4-day-old *Atgpxl* mutants to salt and osmotic stresses for two weeks. Measuring the length of the primary roots of untreated 18-day-old seedlings revealed that the maximal root length of *Atgpxl1* and -*3* was decreased, while *Atgpxl2*, -*5*, and -*7* did not differ significantly from Col-0, but the growth of *Atgpxl4*, -*6*, and -*8* roots increased. Counting the number of visible lateral roots uncovered that *Atgpxl5*, -*6*, and -*8* mutants had more lateral roots than the wild type plants, but the lateral root density was higher only in the *Atgpxl5* roots (Figure 2).

Different concentrations of NaCl had an inverse effect on the lengthwise growth of the wild type roots, where a 50 mM salt concentration promoted root growth while 100 mM inhibited root growth. Among the mutants, the root growth in *Atgpxl6,* -*7*, and -*8* mutants increased compared to Col-0. Interestingly, except for *Atgpxl3*, all mutants had longer roots in the presence of 100 mM NaCl than the wild type plants. However, the number of lateral roots differed significantly from the salt-stressed wild type roots only in the 100 mM NaCl-treated *Atgpxl3* mutant roots, and the density of lateral roots was significantly lower in the 100 mM NaCl-treated *Atgpxl1* mutant (Figure 2a).

The used isosmotic mannitol concentrations decreased both the total length of primary roots and the number of lateral roots in the wild type seedlings after the 14 d treatment, but the density of lateral roots was not affected (Figure 2b). Similar to the effect of the 100 mM NaCl treatment, the shortest root length among the mutants was measured at 200 mM-treated *Atgpxl3*, but all the other mutants had longer roots in the presence of 200 mM mannitol than the wild type plants. The roots of *Atgpxl2*, -*7*, and -*8* mutants grew better than Col-0 in both mannitol treatments. Although significant differences were found only in two mutants in the number of lateral roots, compared to the wild type plants, the lateral root density of *Atgpxl1*, -*2*, -*3*, and -*5* mutants was lower after applying 200 mM mannitol, while in *Atgpxl6*, -*7*, and -*8*, this parameter decreased after both osmotic stress treatments (Figure 2b).

### 3.3. Slightly Elevated ROS Levels Decreased the Vitality of the Mutant Roots

**Figure 3:**
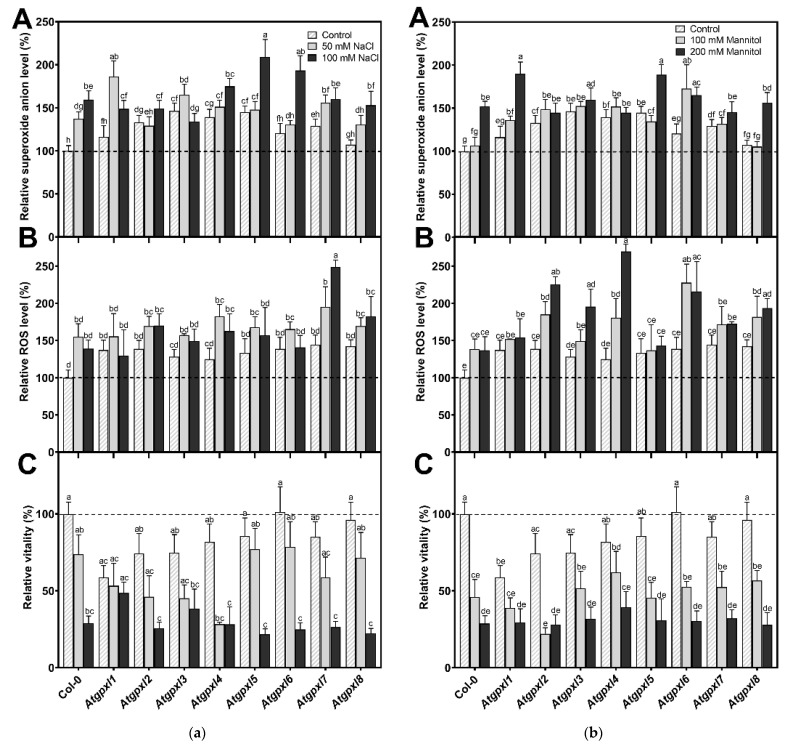
(**a**) The superoxide anion (A), total ROS levels (B), and the intensity of metabolic activity (vitality; C) in the roots of 18-day-old *Arabidopsis* Col-0 and *Atgpxl1*–*8* mutant seedlings. The parameters were detected under control conditions and after two weeks of 50 mM and 100 mM NaCl treatments. The values presented here are the fluorescence intensities in control%, where the control is the untreated Col-0 (mean ± SE, n ≥ 45). (**b**) The superoxide anion (A), total ROS levels (B), and the intensity of metabolic activity (vitality; C) in the roots of 18-day-old *Arabidopsis* Col-0 and *Atgpxl1*–*8* mutant seedlings. The parameters were detected under control conditions and after two weeks of 100 mM and 200 mM mannitol treatments. The values presented here are the fluorescence intensities in control%, where the control is the untreated Col-0 (mean ± SE, n ≥ 45). Dashed lines show the values of the untreated wild type to highlight the differences compared to those data. Data were analyzed using one-way ANOVA followed by Duncan’s multiple range test, and different letters represent data considered statistically significant at *p* ≤ 0.05.

**Figure 4:**
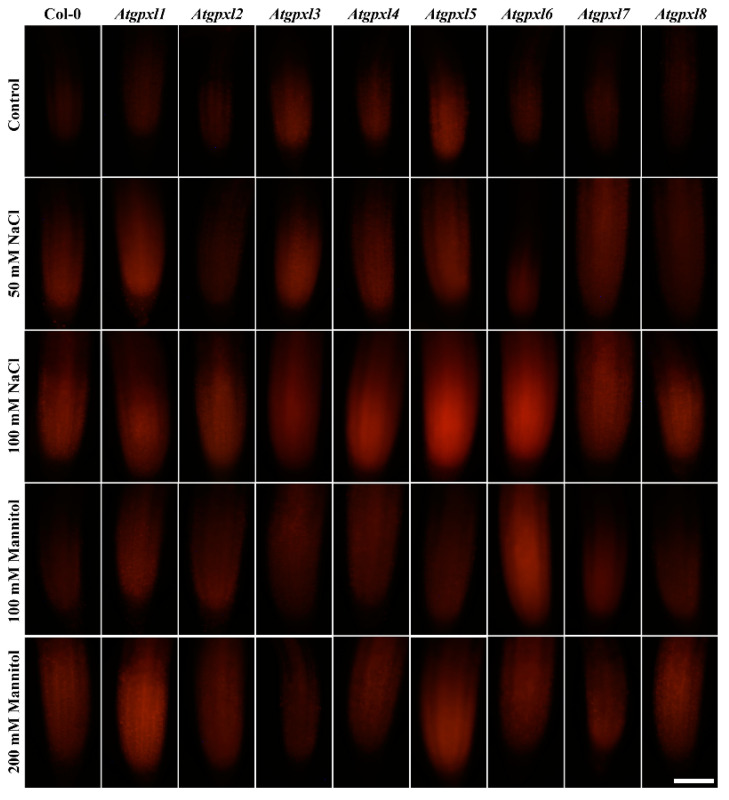
Representative fluorescent microscopy images about the superoxide anion levels (oxyethidium fluorescence) in control and treated (50/100 mM NaCl, 100/200 mM mannitol) roots of 18-day-old *Arabidopsis* Col-0 and *Atgpxl1*–*8* mutant seedlings. The white scalebar is equal to 100 µm.

Using dihydroethidium allows us to investigate the superoxide (O_2_^•−^) levels in roots. The detection of the DHE fluorescence in the non-treated 18-day-old seedlings revealed elevated O_2_^•−^ levels in the roots of *Atgpxl2*, -*3*, -*4*, -*5*, and -*7* seedlings under control conditions (Figure 3 and Figure 4). Supplementation of the media with 50 or 100 mM NaCl increased the O_2_^•−^ level in wild type roots by 37 and 59%, respectively, after 14 days. These treatments usually resulted in a moderate increase in the fluorescence intensity of mutants. Exceptions are the effect of the 50 mM NaCl on *Atgpxl1* and the 100 mM NaCl on *Atgpxl5* and -*6* roots, where ca. 185, 210, and 195% O_2_^•−^ accumulations were detected, respectively, as shown in Figure 3a, as relative percentages to the untreated Col-0 control. On the contrary, the isosmotic 100 mM mannitol did not change the O_2_^•−^ content of the wild type roots and, except for the elevation in *Atgpxl6* roots, this stress did not significantly influence the level of this ROS in the mutants. However, in the presence of 200 mM mannitol, the O_2_^•−^ content of Col-0 increased by ca. 50%. Due to the applied osmotic stress, the O_2_^•−^ accumulation was similar in most of the mutants as well, but it was even higher in the *Atgpxl1* and -*5* roots (Figure 3b and Figure 4), which indicates specific roles of the encoded AtGPXLs in the processing of O_2_^•−^.

**Figure 5:**
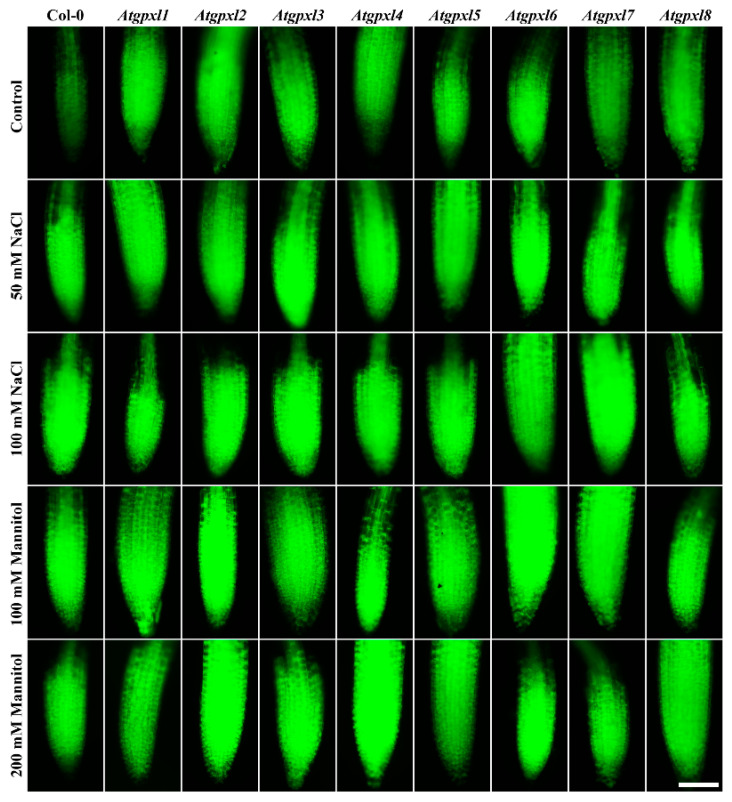
Representative fluorescent microscopy images about the total ROS levels (2′,7′-dichlorofluorescein fluorescence) in control and treated (50/100 mM NaCl, 100/200 mM mannitol) roots of 18-day-old *Arabidopsis* Col-0 and *Atgpxl1–8* mutant seedlings. The white scalebar is equal to 100 µm.

**Figure 6:**
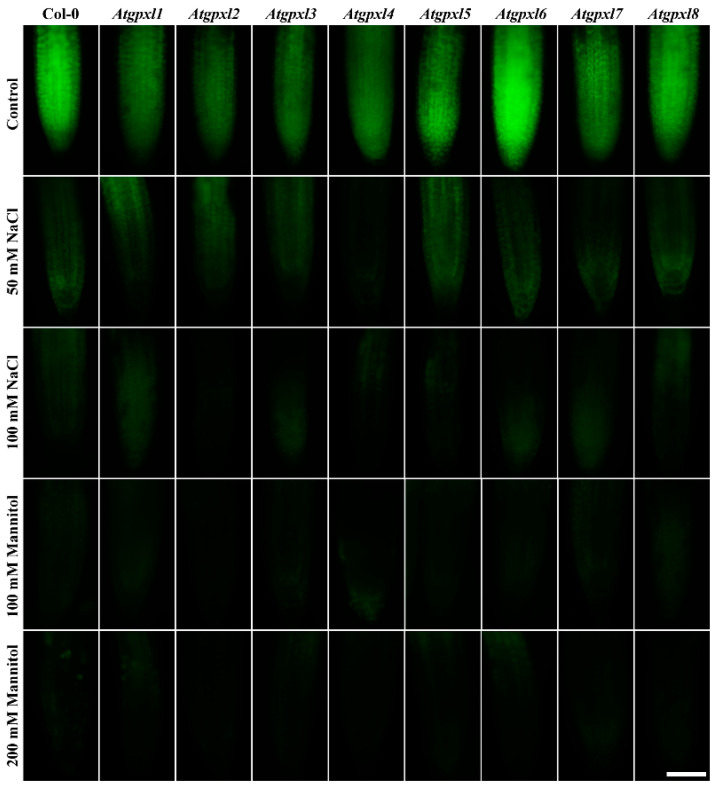
Representative fluorescent microscopy images about the metabolic activity (vitality; fluorescein fluorescence) in control and treated (50/100 mM NaCl, 100/200 mM Mannitol) roots of 18-day-old *Arabidopsis* Col-0 and *Atgpxl1–8* mutant seedlings. The white scalebar is equal to 100 µm.

Detecting the total ROS levels in the roots by H_2_DCFDA staining revealed that untreated *Atgpxl* mutants did not accumulate significantly higher amounts of ROS compared to the wild type plants and, except for the decreased vitality of *Atgpxl1* after the osmotic stress treatment, their vitality was similar to the Col-0 roots (Figure 3, Figure 4, Figure 5 and Figure 6).

The 50 and 100 mM NaCl elevated the total ROS levels of the Col-0 plants by 55 and 39%, respectively, while applying 100 or 200 mM mannitol increased them by ca. 35%, but these changes did not prove to be significant. The vitality of the wild type roots decreased by 26 and 71% due to 50 and 100 mM NaCl treatments, respectively, and the isosmotic 100 and 200 mM mannitol caused a ca. 55 and 70% decrease, respectively. In the mutants, the investigated parameters changed rather similarly to the wild type, with a few exceptions, such as the higher ROS accumulation in *Atgpxl7* roots after treatment with 100 mM NaCl, in *Atgpxl6* after applying 100 mM mannitol and in *Atgpxl2*, -*3*, and -*4* roots after 200 mM mannitol treatments (Figure 3, Figure 4, Figure 5 and Figure 6).

### 3.4. The Leaves of Untreated Mutant Seedlings Accumulated More Total ROS, and Most of Them Showed Less Metabolic Activity

A comparison of the ROS levels of the 18-day-old untreated leaves revealed that the mutation of a single *AtGPXL* gene caused elevated dichlorofluorescein fluorescence in all investigated T-DNA insertional mutants’ shoots compared to Col-0. Parallelly, the vitality detected by FDA decreased in most of the mutants (exceptions are *Atgpxl1* and *Atgpxl2*).

**Figure 7:**
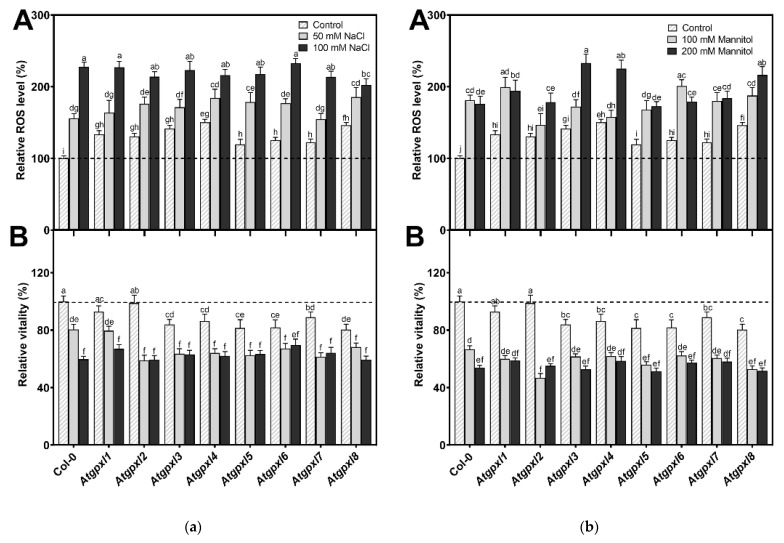
The total ROS level (A) and the intensity of metabolic activity (vitality; B) in the shoots of 18-day-old *Arabidopsis* Col-0 and *Atgpxl1*–*8* mutant seedlings. (**a**) The parameters were detected under control conditions and after two weeks of 50 mM and 100 mM NaCl treatments. (**b**) The parameters were measured after two weeks of 100 mM and 200 mM mannitol treatments. The values presented here are the fluorescence intensities in control%, where the control is the untreated Col-0 (mean ± SE, n ≥ 45). Dashed lines show the values of the untreated wild type to highlight the differences. Data were analyzed using one-way ANOVA followed by Duncan’s multiple range test, and different letters represent data considered statistically significant at *p* ≤ 0.05.

In the presence of 50 and 100 mM NaCl, the total ROS level of Col-0 increased by 56 and 127%, while the vitality decreased by 20 and 40%, respectively. There was no significant difference between the ROS level of *Atgpxl1*–*8* mutants and Col-0 cotyledons after applying 50 mM NaCl treatment, but the vitality of the *Atgpxl2*–*8* mutants was lower than in the wild type. Interestingly, the 100 mM NaCl treatment did not result in any difference in the ROS levels and in the vitality of the seedling’s shoots; moreover, the ROS content in *Atgpxl8* shoots was even lower than in other genotypes (Figure 7a), suggesting that other ROS-processing mechanisms were induced and might ensure similar ROS levels.

The 14 d 100 and 200 mM mannitol treatments elevated the ROS level in the wild type by ca. 80% simultaneously; meanwhile, the vitality decreased by 34 and 46%, respectively. Generally, the ROS and the vitality in the mutants’ leaves changed similarly after mannitol treatment in the presence of isosmotic NaCl concentrations, but more ROS accumulated in the leaves of *Atgpxl3*, -*4*, and -*8* seedlings in the case of 200 mM mannitol. The vitality decreased more in the *Atgpxl2*, -*5*, and -*8* mutants compared to the wild type plants after applying 100 mM mannitol, but there was no difference between the metabolic activity of the seedlings after treatments with a higher concentration of mannitol (Figure 7b).

### 3.5. Correlations Between the Measured Parameters Strengthen the Involvement of AtGPXLs in ROS Homeostasis

**Figure 8:**
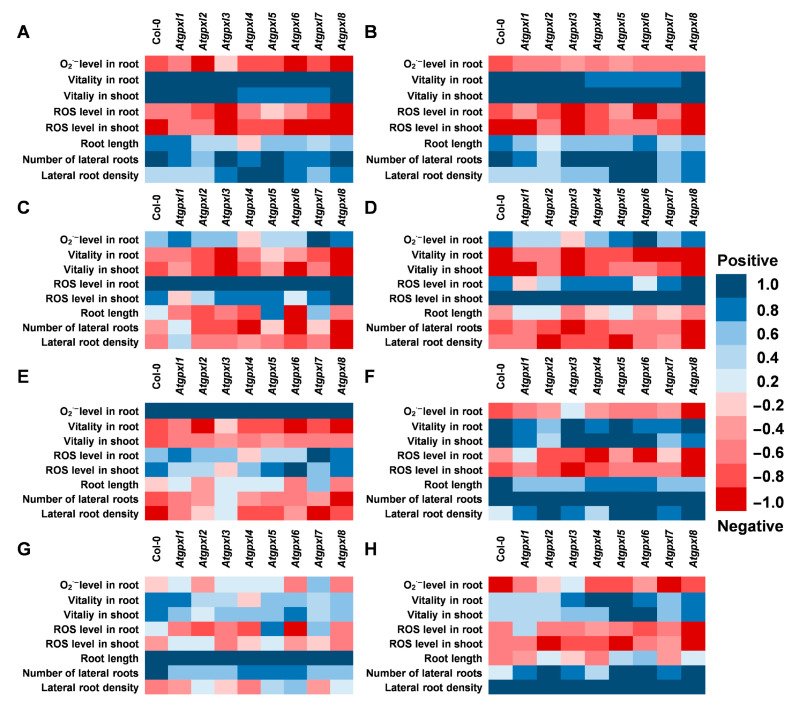
Correlation analysis between the detected and measured parameters (**A**) with the vitality in roots, (**B**) with the vitality in shoots, (**C**) with the total ROS level in roots, (**D**) with the total ROS level in shoots, (**E**) with the superoxide anion level in roots, (**F**) with the number of lateral roots, (**G**) with the total root length, and (**H**) with the lateral root density under control conditions and 2 weeks after applying 50/100 mM NaCl or 100/200 mM mannitol treatments in *Arabidopsis thaliana* wild type (Col-0) and *Atgpxl1*–*8* mutant seedlings. Blue colors show a positive correlation and red colors show a negative correlation, according to the color scalebar.

Since the determination of *E*_GSH_ was performed on 6-day-old seedlings (when the lower number of cell layers allows for a more precise detection of ratiometric roGFP2 fluorescence), the other parameters were measured after two weeks of stress treatments. The correlation analysis was performed on data including the growth parameters, ROS levels, and metabolic activity of 18-day-old seedlings. Our results indicated a very strong positive correlation between the shoot and root vitality, which was in negative correlation with ROS levels in shoots and roots in all plants (Figure 8A,B). However, the correlation between the ROS levels and the vitality in the two organs is not that strong and is even absent in the case of *Atgpxl1*, -*2*, and -*6* (Figure 8C,D). This analysis revealed a stronger negative correlation between the ROS levels of wild type shoots and the development of lateral roots than between the ROS of roots proximal meristematic region and the growth of lateral roots. These correlations altered in the *Atgpxl* mutants; in some cases, they were absent (*Atgpxl1*, -*5*, and -*7*), while in some other mutants (*Atgpxl4*, -*6*, and -*8*), they became stronger (Figure 8E–H).

## 4. Discussion

### 4.1. The ROS-Processing Roles of AtGPXLs in the Mutants Were Not Substituted Completely Either Under Control or Abiotic Stress Conditions

Several reports provided direct or indirect evidence that plant GPXLs may have multiple roles, particularly during abiotic stresses. Chen et al. [49] reported that tomato GPXLs in tobacco plants functioned as cytoprotectors, preventing Bax, heat, salt, and abiotic stress-induced cell death. In addition to the protection of biological membranes by the reduction in lipid peroxides, GPXLs may preserve proteins and DNA in case of oxidative stress [10,11,20]. It was proven that *A. thaliana* plants overexpressing *AtGPXL8* had less oxidized proteins and nucleotides under oxidative stress [20]. It was suggested that their role is more important in the detoxification of peroxides other than H_2_O_2_ [8], or they even might have a crucial role in redox signaling [42]. However, the spectrophotometrically measured H_2_O_2_ level in our previous experiments in *Atgpxl5* mutant plants was higher both under control conditions and in the presence of 100 mM NaCl than in the wild type, while in OX-AtGPXL5 plants, it was on the same level as in untreated Col-0 wild type plants [22]. Our present results confirm that AtGPXLs are involved in the maintenance of ROS homeostasis, and a mutation in one of their coding genes can negatively affect the vitality of seedlings. Interestingly, the highest differences among the O_2_^•−^ and total ROS levels of wild type and *Atgpxl1*–*8* mutants were found usually under control conditions or after applying the lower concentrations of salt and osmotic stress (50 mM NaCl and 100 mM mannitol, respectively). This indicates a rather special role of AtGPXLs since probably other ROS-processing mechanisms are responsible for maintaining the vitality of these mutants under more severe stresses.

It is worth noting that the produced H_2_O_2_ can be reduced by catalase and other peroxidases as well. In the maintenance of ROS homeostasis, by the elimination of H_2_O_2_ and organic hydroperoxides, the function of *Arabidopsis* GPXLs shows some overlaps with peroxiredoxins and glutathione transferases (GSTs) with peroxidase activity [10,11]. Measuring the non-enzymatic antioxidants in the 6-week-old hydroponically grown *Atgpxl* mutants revealed altered ascorbic acid contents in shoots and increased GSH levels, particularly in the roots [44]. Moreover, it is also possible that the deficiency of one GPXL isoenzyme could be compensated by other GPXLs, as was found in the case of *Arabidopsis* tau group GSTs [48]. Even so, after two weeks of 100 mM NaCl or 200 mM mannitol treatments, the elevated ROS levels and decreased metabolic activity in several mutant seedlings indicate their importance as antioxidant isoenzymes, which have specific roles in ROS processing or redox signaling under control conditions and mild stress. Whether their primary effect is a direct antioxidant function or the modification of antioxidant systems through involvement in redox signaling requires further investigation; for example, by estimating the very early stress responses.

### 4.2. AtGPXLs Have Specific Functions in Tissues and Organs

Phylogenetic analysis of *Arabidopsis* GPXL protein sequences revealed that the eight isoenzymes can be grouped into four pairs that show stronger similarity with each other than with the rest of the family. These are the AtGPXL1 and AtGPXL7, AtGPXL2 and AtGPXL3, AtGPXL4 and AtGPXL5, and AtGPXL6 and AtGPXL8 [8,13]. Detailed analysis of their subcellular localization showed that AtGPXL1 and -7 are chloroplastic enzymes, AtGPXL2 and -8 exhibited cytosolic-nuclear localization, AtGPXL3 is a luminal protein that can be bound to the ER and Golgi membranes, AtGPXL4 and -5 are anchored to the plasma membrane, and AtGPXL6 can be found mainly in mitochondria [13]. Interestingly, their involvement in different growth or developmental processes was also reported, and their expression depends on tissues and developmental stages (e.g., [10,11,15]). Analysis of microarray data has shown that *AtGPXL2*, -*3*, and -*8* were highly upregulated during germination, while *AtGPXL1*, -*4*, -*5*, -*6*, and -*7* were downregulated. In the seedling stage, *AtGPXL3* showed the highest expression in the shoot apical meristem, but a rather high expression was reported in the case of *AtGPXL1*, -*2*, and -*7* or -*8* in the shoot tip and cotyledon and generally in the leaf tissues [8]. In the roots, the *AtGPXL8* was expressed at the highest level in the radicle, but the transcript amounts of *AtGPXL2*, -*3*, -*5*, and -*6* were also rather high both in the seedling stage and later on. However, their expression patterns depended on the root zones [8]. Focusing on the growth of roots, in line with the findings in the literature, the length of primary roots and/or the number of lateral roots has changed significantly in the untreated *Atgpxl2*, -*5*, -*6*, and -*8* mutants in the present experiments (Figure 2). Our correlation analysis revealed a higher negative correlation between the ROS levels and the numbers and growth of lateral roots compared to the found correlation in the wild type plants (Figure 7). However, this correlation was significantly disturbed in most *Atgpxl* mutants. Because auxin is the key hormone of lateral root initiation, this may further confirm the relationship between GPXL proteins and auxin.

It was reported that H_2_O_2_ has a role in root hair formation and the inhibition of root growth, while O_2_^•−^ is necessary for root elongation [2,50]. The main sources of O_2_^•−^ production are the electron transport systems and different enzymatic mechanisms, like the activity of NADPH oxidase, xanthene dehydrogenase, and aldehyde oxidase [51]. Although O_2_^•−^ might convert to H_2_O_2_ even spontaneously at low pH, the role of SOD isoenzymes has high importance in the process, and they can be regarded as the first line of the antioxidant defense [52] or can act as a member of the ROS signaling route. Interestingly, it was suggested that GPXL2 can be part of a protein complex involved in O_2_^•−^ conversion that can be activated under stress [8,53], but as far as we know, this theory has not been proven yet. Even if probably there are no direct connections between the GPXL enzymes and O_2_^•−^, the O_2_^•−^ concentrations were significantly higher in *Atgpxl2* and in more than half of the mutants in our experiments than in the wild type plants. This difference was noticeable not only under control conditions but also in the case of certain mutants (*Atgpxl5*, -*6*) during stress.

It has been suggested that the primary peroxide substrates of plant GPXLs are organic peroxides rather than H_2_O_2_. Using fluorescent dyes and microscopic methods, we demonstrated that total ROS levels and superoxide anion levels are generally elevated in both cotyledons and roots. Since this was observed under control conditions as well as after mild salt stress, our results underscore the importance of these enzymes even under normal physiological conditions.

### 4.3. AtGPXLs Are Related to Redox Signaling

Increased ROS production temporarily shifts the redox balance toward a more oxidized state, which can be interpreted either as an active acclimation (adaptation) response or as damage [3,54]. Several reports indicated the involvement of GPXLs in redox signaling mechanisms; moreover, their mediating role in the crosstalk between GSH and TRX pathways was suggested [12,24,26,41,42,43,55]. There is increasing evidence that these redox components are involved in healthy plant growth, development, successful organogenesis, and the regeneration of cultured cells [4,12,56,57]. The *Arabidopsis root meristemless1* (*rml1*) mutant, which has a defect in GSH biosynthesis [58], is unable to maintain the root apical meristem; however, the apical meristem in its shoot is not affected, possibly because of the TRX-dependent control [59]. The characterization of the *rml1* mutant discovered an altered expression of several hundred genes, among them numerous encode redox-related proteins, such as glutaredoxins (GRXs), h-type thioredoxins (TRXhs), and GPXLs [26].

Thiol redox biochemistry is considered to have a fundamental role in cellular processes, even in the growth of cells. GSH can alter the expression of genes through the modulation of the redox state of proteins and transcription factors and is thought to be the main redox regulator, but this mechanism is yet poorly understood [60]. Glutathione limits the lifetime of the oxidative signals because of its antioxidant function, and the maintenance of a high GSH level and the GSH/GSSG ratio is important in the proper function of the cells and organs [59]. In general, the GSH-dependent redox system is regarded to be a housekeeping redox system, and *E*_GSH_ is a relatively constant parameter in the plant cytosol [34]. Interestingly, measuring the GSH and GSSG amounts in 6-week-old hydroponically grown *Atgpxl* mutants revealed elevated GSH content and a more reduced redox state compared to the wild type [44]. Our other results indicated that the key mechanism of AtGPXL5 might be the modification of the redox status [22]. The overexpression of *AtGPXL5* increased the amount of total GSH; thus, the glutathione redox potential became more negative than in the wild type. These changes were statistically significant under control conditions only in the shoots, but 24 h treatment with 100 mM NaCl caused significant differences in the roots as well. However, the elevated peroxide levels of the *Atgpxl5* mutant compared to the wild type indicated that AtGPXL5 has a role in fine tuning ROS homeostasis [22]. Among the 6-day-old untreated *Atgpxl1–8* seedlings, we found the largest changes in *E*_GSH_ (approximately 25 mV) in the leaves of *gpxl1* and -*7* seedlings, demonstrating their importance in the maintenance of redox homeostasis. Furthermore, these results provide additional insights into the significance of AtGPXLs in various physiological states.

It is widely accepted that environment-responsive developmental plasticity is linked to ROS and enzymatic and non-enzymatic antioxidants, and is in strict relationship with hormonal control in the development of the roots [1,2,61,62,63]. It was reported that the altered ROS levels and redox state influenced the growth of the proximal meristem of 7-day-old *Arabidopsis* roots [35]. Using the roGFP1 redox sensor, it was demonstrated that the redox profile of 3–9-day-old *Arabidopsis* roots shifted toward the more oxidized state due to 50–150 mM NaCl treatments at the early stage of stress, but it was re-established after 6 days [33]. Moreover, these authors described the connection between the changes in *E*_GSH_ and auxin transport using in vivo redox probes [33].

Changes in the redox profile of the root tissues, caused by salt treatments, influenced the patterns of both PIN1 and PIN2 auxin efflux carriers and the AUX1 influx carrier, therefore changing the root meristem size [33]. Since GPXLs are involved in forming the redox status of plants, they may also affect hormone transport, metabolism, and signaling as well. The relevance of AtGPXL7 in hormone-mediated root development, especially in lateral root development, was also demonstrated by 1-naphtaleneacetic acid and synthetic strigolactone treatments [24]. The interaction of auxin and ROS in plant growth and development has a role under salt stress conditions to trigger dynamic responses [63,64]. The elevated ROS production negatively modulates root growth by regulating the PIN-mediated auxin polar transport in the roots, and it affects root meristem activity [64]. Auxin may alter root growth and development through modulating the redox balance, e.g., by the regulation of APX activity [65,66], and enhanced redox metabolism was reported in the auxin receptor mutant *tir1afb2* that showed enhanced tolerance to salt stress [67].

In our present study elevated ROS levels were found after both the NaCl and mannitol treatments, especially in shoots, and the correlation analysis revealed a higher negative correlation between ROS levels and the number and growth of lateral roots, instead of the growth parameters of the primary roots in the wild type plants. However, this correlation was significantly altered in the mutants (Figure 7). The relationship between the activation of GPXL enzymes and the triggered hormonal changes needs further investigation. Our previous experiments uncovered the phenotypical effects of the mutation and overexpression of *AtGPXL5* under NaCl stress; also, the interaction between AtGPXLs and ethylene signaling was demonstrated [68]. Our present correlation analysis indicates the primary role of ROS-dependent effects of AtGPXLs in altering the development of seedlings and in response to abiotic stresses. The isoenzyme-specific pattern highlighted that AtGPXLs having distinct interactions with other pathways are integrated members of the signaling network.

## 5. Conclusions

AtGPXLs play a fine-tuning role in regulating ROS levels in seedlings under both control conditions and after two weeks of abiotic stress. Introducing the genetically encoded roGFP2 redox probe into *Arabidopsis* wild type and mutant plants allowed us to demonstrate in vivo that the redox status of most untreated mutant seedlings is more oxidized than that of the wild type.

The importance of these enzymes under normal and mild stress conditions is likely linked to redox signaling. While other antioxidant mechanisms may be activated under severe stress, they appear insufficient to fully suppress ROS levels. Consequently, mutations in an *AtGPXL* gene can alter the overall cellular redox status. We still cannot determine whether this is due to their direct ROS-scavenging activity or whether they act indirectly, for example, through the redox regulation of ROS-processing mechanisms, including other enzymatic or non-enzymatic systems. Their significance may be linked to redox signaling pathways that impact the thiol/disulfide balance. Since roGFP2 monitors the redox state of glutathione, our results also suggest that GSH plays a more crucial role in AtGPXL function than previously assumed. Hormones such as auxin may also exert their effects in a redox-dependent manner, with AtGPXLs potentially contributing to this process.

Our correlation analysis reinforces the role of GPXL enzymes in supporting plant growth and development by modulating ROS levels in an organ-specific manner. The observed differences in root phenotype and metabolic activity of *Atgpxl* mutant plants reflect the complex interplay between AtGPXLs, redox homeostasis, and plant hormones.

Uncovering the key regulatory networks, their components, and the nature of their interactions still requires further intensive research.

## Figures and Tables

**Table 1:** The redox potential of roGFP2 (mV) in the cotyledons and root tips (meristematic region) of 6-day-old *Arabidopsis* Col-0 and glutathione peroxidase mutant (*gpxl1*–*8*) seedlings expressing the cytosolic redox probe. Mean ± SD, n ≥ 3. Columns marked with different letters are significantly different from each other at a probability level of *p* ≤ 0.05 (Duncan’s test).

Genotype	Cotyledon	Root
Col-0	−302.27 ± 4.66 ^d^	−302.48 ± 5.88 ^c^
*gpxl1*	−274.25 ± 11.92 ^a^	−269.30 ± 1.02 ^a^
*gpxl2*	−294.59 ± 2.76 ^c^	−289.84 ± 4.01 ^b^
*gpxl3*	−285.03 ± 3.06 ^b^	−296.69 ± 1.76 ^c^
*gpxl4*	−298.93 ± 6.89 ^cd^	−271.86 ± 9.25 ^a^
*gpxl5*	−298.46 ± 4.96 ^cd^	−298.77 ± 4.30 ^c^
*gpxl6*	−287.90 ± 6.88 ^b^	−278.71 ± 8.39 ^a^
*gpxl7*	−277.97 ± 3.36 ^a^	−300.50 ± 0.89 ^c^
*gpxl8*	−284.01 ± 8.28 ^ab^	−281.32 ± 6.43 ^ab^

## Data Availability

The data presented in this study are openly available in Zenodo at https://doi.org/10.5281/zenodo.14930740.
